# The New Nematicide Cyclobutrifluram Targets the Mitochondrial Succinate Dehydrogenase Complex in *Bursaphelenchus xylophilus*

**DOI:** 10.3390/ijms25136914

**Published:** 2024-06-24

**Authors:** Wenyi Liu, Hudie Shao, Danni Qi, Xiaofang Huang, Jing Chen, Lifeng Zhou, Kai Guo

**Affiliations:** College of Forestry and Biotechnology, Zhejiang A&F University, Hangzhou 311300, China; liuwy@stu.zafu.edu.cn (W.L.);

**Keywords:** *Bursaphelenchus xylophilus*, trunk injection, cyclobutrifluram, nematocidal activity, succinate dehydrogenase inhibitors

## Abstract

*Bursaphelenchus xylophilus* is a dangerous quarantine pest that causes extensive damage to pine ecosystems worldwide. Cyclobutrifluram, a succinate dehydrogenase inhibitor (SDHI), is a novel nematicide introduced by Syngenta in 2013. However, the nematocidal effect of cyclobutrifluram against plant-parasitic nematodes remains underexplored. Therefore, here, we aim to address this knowledge gap by evaluating the toxicity, effects, and mode of action of cyclobutrifluram on *B. xylophilus*. The result shows that cyclobutrifluram is the most effective agent, with an LC_50_ value of 0.1078 mg·L^−1^. At an LC_20_ dose, it significantly reduced the population size to 10.40 × 10^3^ ± 737.56—approximately 1/23 that of the control group. This notable impact may stem from the agent’s ability to diminish egg-laying and hatching rates, as well as to impede the nematodes’ development. In addition, it has also performed well in the prevention of pine wilt disease, significantly reducing the incidence in greenhouses and in the field. SDH consists of a transmembrane assembly composed of four protein subunits (SDHA to SDHD). Four *sdh* genes were characterized and proved by RNAi to regulate the spawning capacity, locomotion ability, and body size of *B. xylophilus.* The mortality of nematodes treated with *sdhc*-dsRNA significantly decreased upon cyclobutrifluram application. Molecular docking further confirmed that SDHC, a cytochrome-binding protein, is the target. In conclusion, cyclobutrifluram has a good potential for trunk injection against *B. xylophilus.* This study provides valuable information for the screening and application of effective agents in controlling and preventing PWD in forests.

## 1. Introduction

Pine wilt disease (PWD) is one of the most dangerous diseases in global forestry ecosystems, which is caused by the nematode *Bursaphelenchus xylophilus* (pine wood nematode, PWN) [1]. *B. xylophilus* develops from an egg to an adult through three propagative larval stages (the second-stage juveniles (J2), J3, and J4). The J1 stage molts to J2 within the egg. Under harsh conditions, *B. xylophilus* molts from J2 to the third stage dispersal juvenile (D3), then to D4 in the presence of the vector beetle. The D4 nematodes are carried to new healthy host trees, completing their life cycle [2]. It has resulted in devastating effects on the pine forests across Asia and Europe over the past century. Tree trunk injection with nematicides is a primary method for controlling PWD, leveraging the transpiration process of the trees to distribute the agents throughout affected areas [3]. Abamectin [4] and emamectin benzoate [5] are commonly used for this purpose, targeting the GABA receptor and glutamate-gated chloride channels (GluCl) in invertebrates to paralyze nematodes [6]. The repeated use of these nematicides can create selection pressure, increasing the risk of developing drug-resistant strains of PWN [7,8]. There is a pressing need to develop new nematicides and replace current treatments to manage PWD more effectively.

Succinate dehydrogenase inhibitors (SDHIs) represent a rapidly evolving class of pesticides targeting SDH. Possessing spectral inhibitory properties, SDHIs are extensively employed for fungal [9,10], acarid [11], and nematode [12] control. SDH comprises a transmembrane assembly composed of four protein subunits (SDHA to D), a flavin adenine dinucleotide (FAD) cofactor (associated with SDHA), three iron–sulfur clusters (housed within SDHB), and a heme moiety (positioned between SDHC and D). SDHA (flavin protein, FP) and SDHB (iron sulfide protein, IP) constitute the soluble components of complex II, exhibiting succinate dehydrogenase function. In contrast, SDHC and SDHD are membrane-integrated proteins pivotal for immobilizing FP and IP within the inner mitochondrial membrane, governing ubiquinone reductase activity. Lipid-soluble redox-active quinones at the Q site expedite electron and proton transport across the respiratory membrane complexes. Succinate dehydrogenase inhibitors bind to the ubiquinone pocket (Q site) within SDH (comprising SDHB, SDHC, and SDHD subunits), thereby impeding respiration via competitive antagonism [9,13]. This inhibition disrupts the conversion of succinate to fumarate and the reduction of ubiquinone to panthenol. Notably, the crystal structure of SDH has been elucidated in Escherichia coli, chickens, and pigs [9].

Cyclobutrifluram, a novel SDHI nematicide developed by Syngenta, features a unique carboxamide structure with a four-membered ring, offering a new approach to nematicide development [14]. To date, limited research has been conducted on the effectiveness of cyclobutrifluram. A study on *Caenorhabditis elegans* indicated a significant reduction in germ cell quantity, pointing to potential impacts on survival and fertility [15]. This nematicide operates by impeding the mitochondrial SDH complex, similar to the mechanism of fluopyram, a well-studied nematicide effective against *B. xylophilus* [16,17]. With its analogous mechanism, cyclobutrifluram is anticipated to exhibit effectiveness in combating *B. xylophilus*. Therefore, our objectives were as follows: (a) To assess the toxicity of cyclobutrifluram against *B. xylophilus*, along with abamectin, emamectin benzoate, and fluopyram; (b) To investigate the effects of these agents on the reproduction, growth, and development of *B. xylophilus*; (c) To evaluate the efficacy of cyclobutrifluram in controlling PWD in greenhouse and field settings; (d) To characterize four *sdh* genes and their functions in *B. xylophilus*; (e) To confirm that cyclobutrifluram targets the SDHs in *B. xylophilus*. This study offers significant insights into the nematocidal activity of cyclobutrifluram against *B. xylophilus*, providing a good basis for the development of new drugs against *B. xylophilus* and for understanding the principle of control targeting SDH.

## 2. Results

### 2.1. Toxicity Test of Four Nematicides against B. xylophilus

Based on the correlation between various concentrations of nematicide and the corresponding mortality rates (Table S1), regression equations and median lethal concentrations (LC_50_) for each agent were determined (Table 1). All agents showed toxicity toward *B. xylophilus*, with cyclobutrifluram demonstrating the highest toxicity (LC_50_ = 0.1078 mg·L^−1^, followed by emamectin benzoate and abamectin with 0.2783 mg·L^−1^ and 1.3874 mg·L^−1^, respectively). The toxicity of fluopyram to *B. xylophilus* was the lowest, with an LC_50_ value of 1.4867 mg·L^−1^.

### 2.2. Effect of Four Nematicides on Reproduction

**Figure 1:**
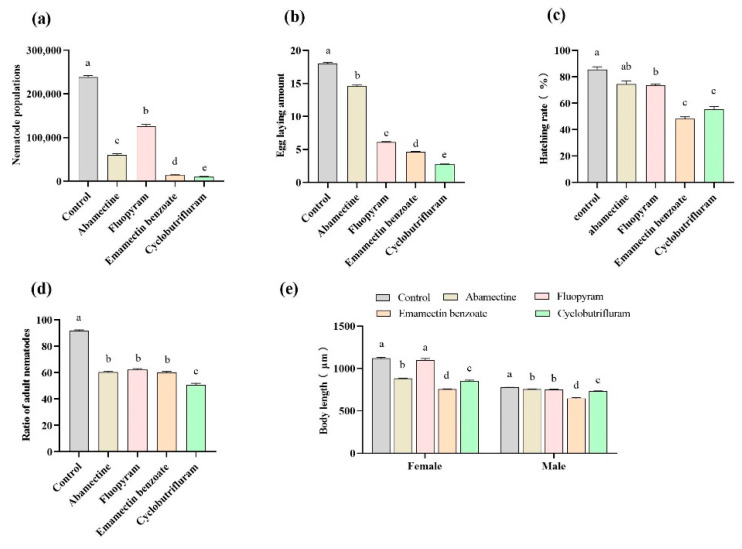
Effects of the four nematicides with LC_20_ concentration against *B. xylophilus*. (**a**) Reproduction, the number of ten nematode pairs, and their offspring within 8 days; (**b**) Egg deposition, the average number of eggs laid by each female when treated with nematicides for 36 h. (**c**) Egg hatch rate, the rate of *B. xylophilus* eggs hatching after being incubated in the nematicide solution for 36 h. (**d**) Development progress, the sexual maturity rate of 100 J2 cultured in *B. cinerea* for 68 h, after they were treated by agents for 24 h. (**e**) Body lengths of offspring whose parents were treated by 0.5 mL agents for 24 h in adult period. Each value represents the mean ± SE of three experiments with three replicates. The different letters on the bars represent significant differences (*p* < 0.05).

In the control group, it took 8 days for 10 pairs of nematodes and their offspring to consume the mycelia of *B. cinerea*. However, the mycelia of *B. cinerea* were still present in the four treatment groups. The effects of the several nematicides on population numbers are displayed in Figure 1a. All of the agents inhibited the reproduction of *B. xylophilus* even at LC_20_ dosages. Among the treated groups, the population sizes (mean ± SE) of *B. xylophilus* were significantly different. In general, the number of offspring in the treated groups was significantly smaller than that in the control group (238.40 × 10^3^ ± 3519.09; *p* < 0.05). Among treatment groups, the cyclobutrifluram-treated group (10.40 × 10^3^ ± 737.56) exhibited the lowest population size, at approximately 1/23 of that in the control group. The medium size was detected in the abamectin-treated (60.40 × 10^3^ ± 2936.67) and emamectin benzoate-treated (14.40 × 10^3^ ± 1193.32) groups. The most offspring were observed in the fluopyram-treated group (126.20 × 10^3^ ± 3841.35).

### 2.3. Effect of Four Nematicides on Egg Deposition

Ten pairs of male and female nematodes were mixed and incubated in the agent at LC_20_ concentration for 36 h. The average number of eggs laid by each *B. xylophilus* female is summarized in Figure 1b. The number (mean ± SE) in all treatment groups was significantly lower than the eggs laid in the control group (18.00 ± 0.34; *p* < 0.05). The number of eggs was significantly different among the treatment groups. In the cyclobutrifluram-treated group, eggs were the least (2.80 ± 0.09), which is only 1/6 of those in the control group. The number of eggs in the other groups was as follows: abamectin (14.60 ± 0.24); fluopyram (6.10 ± 0.16); and emamectin benzoate (4.6 ± 0.16).

### 2.4. Effect of Four Nematicides on Egg Hatching Rate

After incubating *B. xylophilus* eggs in the nematicide solution at LC_20_ concentration for 36 h (Figure 1c), all treatment groups, except those treated with abamectin (74.34 ± 2.47%), had a significantly reduced egg hatching rate (mean ± SE) relative to that of the control group (85.43 ± 2.10%; *p* < 0.05). No differences were observed between the abamectin- and fluopyram-treated (73.48 ± 0.99%) groups. The egg hatching rate of the emamectin benzoate-treated group (48.31 ± 1.36%) was the lowest; it also had a significant difference with other treatment groups, except the cyclobutrifluram-treated group (55.43 ± 2.01%).

### 2.5. Effect of Four Nematicides on Development Progress

Synchronized J2 nematodes were treated with the agents at LC20 concentrations for 24 h and washed and reared on *B. cinerea* in PDA culture plates for 68 h. In the control group, 91.68 ± 0.70% of nematodes reached sexual maturity (mean ± SE) (Figure 1d). In contrast, only approximately 60% of nematodes reached sexual maturity in the abamectin- (60.29 ± 0.94%), fluopyram- (62.16 ± 0.91%), and emamectin benzoate-treated (60.24 ± 0.68%) groups, significantly lower than those in the control group. The cyclobutrifluram-treated group had the lowest sexual maturity rate (50.73 ± 1.12%), which was significantly different from the other treatment groups (*p* < 0.05).

### 2.6. Effect of Four Nematicides on Body Lengths in Offspring

Parental adults were treated with the agents at LC_20_ concentration for 24 h. After mating to produce offspring, the body lengths of the offspring were measured. In the F1, the body lengths (mean ± SE) of females treated with abamectin (883.29 ± 4.75 μm), emamectin benzoate (756.08 ± 4.24 μm), and cyclobutrifluram (853.94 ± 8.99 μm) were significantly shorter than those of the females treated with distilled water (1120.28 ± 9.94 μm) (*p* < 0.05; Figure 1e). Additionally, the length of males treated with agents was significantly shorter than the length of the males in the control group (777.21 ± 2.38 μm) (*p* < 0.05; Figure 1e). The length of males treated with emamectin benzoate (648.31 ± 8.04 μm) was the shortest, and this was followed by cyclobutrifluram (732.39 ± 4.11 μm).

### 2.7. Control Efficacy of Cyclobutrifluram to B. xylophilus in the Greenhouse

**Figure 2:**
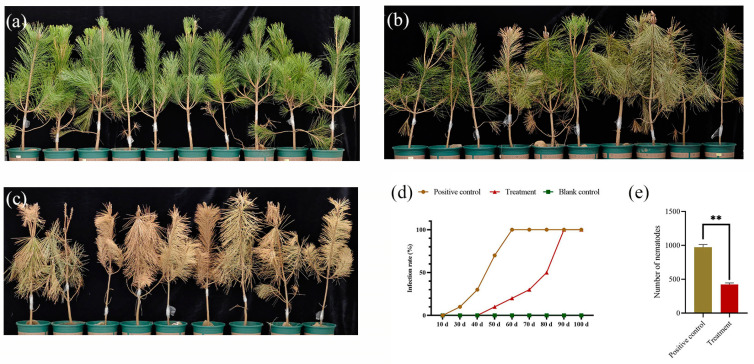
Control efficacy of cyclobutrifluram against *B. xylophilus* in the greenhouse. Representative photographs of the inoculation assay after 90 days: (**a**) Seedlings inoculated with distilled water without nematode in the blank control group; (**b**) Seedlings inoculated with cyclobutrifluram-treated nematodes (0.03 mg/mL) in the treatment group; (**c**) Seedlings inoculated with wild-type nematodes in the positive control group; (**d**) Infection rate of pine trees in different groups over time, with the horizontal axis representing the duration of treatment. (**e**) Number of nematodes per gram of pine branches. Ten repetitions per group. The values represent the mean ± SE of ten replicates and were analyzed by *t*-test. Asterisks indicate statistically significant differences (** *p* < 0.01).

In the blank group (Figure 2a), trees inoculated with distilled water were always green. In the positive control group (Figure 2c), pine trees inoculated with approximately 4000 nematodes gradually turned yellow and withered; it took 60 days for nematodes and their offspring to infect black pine trees to death. At 90 days after inoculation, all the needles turned brown, and the entire tree wilted. However, in the treatment group (Figure 2b), the withered appearance of the trees occurred slowly in seedlings inoculated with nematodes treated with cyclobutrifluram. At 90 days after inoculation, the infection rate of pine trees in the treatment group was significantly lower than that of seedlings inoculated with WT nematodes. A few needles turned yellow, but most of the trees had already been infected and discolored (Figure 2b). After *P. thunbergii* had been infected for 100 days, *B. xylophilus* was detected in both the treatment and positive control groups. The number of nematodes per gram of pine branches in the cyclobutrifluram-treated group was significantly lower than that in the positive control group. This suggests that cyclobutrifluram is effective in reducing the number of *B. xylophilus*, likely exhibiting strong nematicidal activity.

### 2.8. Preventative Effect against the Pine Wilt Disease under Field Conditions

In the infected area, we monitored the infection of healthy *P. massoniana* across different treatment groups over a period of two years. The mortality rate in the control group (mean ± SE %) continued to increase to 26.52 ± 2.74 (Table 2). Treatment with emamectin benzoate at a dosage of 20 mg·cm^−1^ DBH resulted in a reduced mortality rate of 2.14 ± 1.01%. Cyclobutrifluram treatment was more effective, lowering the mortality rate to 1.27 ± 1.34% at a dosage of 10 mg·cm^−1^ DBH. At a higher concentration of 20 mg·cm^−1^ DBH, none of the treated trees died after a period of 24 months. All the pieces of dead pine trees had microscopic detection of *B. xylophilus*.

### 2.9. Phylogenetic Analysis, Model Building, and Molecular Docking of SDHs

**Figure 3:**
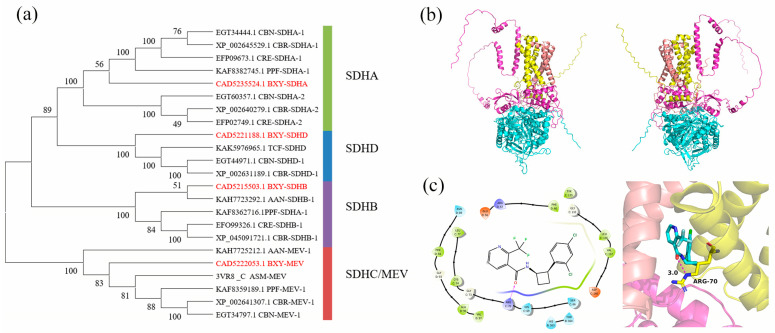
(**a**) The phylogenetic tree of deduced BXY-SDHs compared to other known SDHs of different species. BXY, *B. xylophilus*; CBN, *C. brenneri*; CBR, *C. briggsae*; CRE, *C. emanei*; PPF, *Pristionchus pacificus*; AAN, *Aphelenchus avenae*; ASM, *Ascaris suum*; TCF, *Trichostrongylus colubriformis*. Homology model of *B. xylophilus* succinate dehydrogenase: (**b**) four subunit models were assembled and merged into an SDH model and its flip horizontal model. The four proteins, SDH-A to D, are represented by blue, magenta, yellow, and salmon α-helix; (**c**) Binding modes of cyclobutrifluram with SDH: 2D (**left**); and 3D (**right**). The red arrow points to the binding site. Cyclobutrifluram-Arg70 is represented as blue cartoon and sticks. The dotted line in purple is the bonding hydrogen bond.

A phylogenetic analysis of the four SDH subunits of *B. xylophilus* and seven other species was constructed, demonstrating that BXY-SDHs had the highest level of similarity with other known SDHs (Figure 3a). Then, the four subunits, *B. xylophilus* SDHA to D, were assembled and merged into an SDH model (Figure 3b). The SDH-autodocking showed that cyclobutrifluram formed hydrogen bonds with Arg70 in SDHC at a distance of 3.0 Å (Figure 3c right), which revealed the sites of action of the agent on *B. xylophilus*.

### 2.10. The Expression Levels and Functions of B. xylophilus sdh Genes

**Figure 4:**
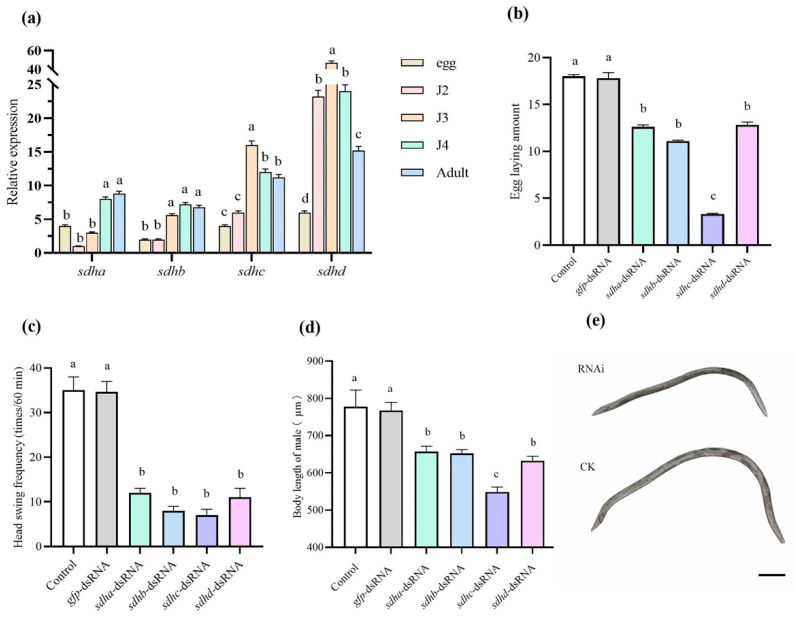
The expression and function of *B. xylophilus sdh* genes. (**a**) Expression levels of four *sdh* mRNA in different development stages. RNAi four *B. xylophilus sdh* genes. (**b**) Egg deposition and the average number of eggs laid by each female in 36 h after being treated with dsRNA. (**c**) The head swing frequency per 1 min of hatched J2 from RNAi eggs. Ten nematodes were counted in each group. (**d**) Body lengths of males who developed from *sdh*-dsRNA-treated eggs. Ten males were counted in each group. (**e**) The male body lengths decreased significantly after treatment with *sdhc*-dsRNA; the scale bar 100 μm. Each value represents the mean ± SE of three replicates. The different letters on the bars represent significant differences (*p* < 0.05).

The qPCR results showed that *sdhd* had the highest expression levels, followed by *sdhc*. The two genes in each developmental stage of *B. xylophilus* had the same trend: the lowest expression in the embryo; the highest expression in the J3 stage. The relative expression levels of *sdha* and *sdhb* were lower among the four *sdh* genes. They all have a higher expression in the J4 and adult stages, with lower expression in the embryo and J2 stages (Figure 4a).

The average number of eggs laid per female over a 36-h period following a 24-h dsRNA treatment was calculated. The number (mean ± SE) in all RNAi groups was significantly lower than the eggs laid in the blank control group (17.80 ± 0.75) and exogenous control group (16.00 ± 0.65) (Figure 4b). In the *sdhc*-dsRNA-treated group, eggs were the least (3.08 ± 0.11), which was significantly different compared with other treatment groups. The number of eggs in the other groups was as follows: *sdha* (12.60 ± 0.40); *sdhb* (11.10 ± 0.21); and *sdhd* (12.78 ± 0.63) (Figure 4b). After incubation, the head swing frequency per minute of J2 was significantly reduced to 7 to 10 times after RNAi compared with the blank control group (35 ± 3) and exogenous control group (34.2 ± 0.35) (Figure 4c). Moreover, RNAi significantly reduced the body lengths of males, especially in the *sdhc*-dsRNA-treated group (Figure 4d,e). Compared with the blank control group (777.21 ± 45 μm) and exogenous control group (764.3 ± 22 μm), the male body length in each treatment group was as follows: *sdha* (656.70 ± 14.40); *sdhb* (652.11 ± 10.34); *sdhc* (548.31 ± 13.21); and *sdhd* (632.39 ± 12.00).

### 2.11. RNAi of sdh Decreases Cyclobutrifluram Susceptibility in B. xylophilus

**Figure 5:**
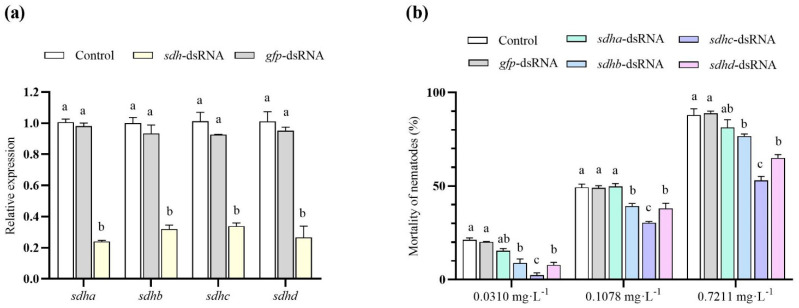
(**a**) RNAi efficiency of four *sdh*-dsRNA in *B. xylophilus* and (**b**) mortality of *B. xylophilus*. The mixed-age *B. xylophilus* were collected and treated by four *sdh*-/*gfp*-dsRNA or ddH_2_O for 24 h. The gene expression level of blank control group was set to one. Mortality was evaluated after nematodes in each group were exposed to LC_20_, LC_50_, LC_90_ (0.031, 0.1078, 0.7211 mg/L) of cyclobutrifluram for 24 h. Different lowercase letters indicate significant differences (*p* < 0.05). Mean ± SE bars are represented by three replicates.

Mixed-age *B. xylophilus* were treated with *sdh*-dsRNA for 24 h, and the gene expression level of *sdh* was measured using RT-qPCR. Compared with the blank control and exogenous control groups, the expression of *sdh* in the dsRNA-treated group was significantly decreased (Figure 5a). The gene expression level of *sdha*, *b*, *c*, and *d* decreased by 76%, 69%, 66%, and 73%, respectively, after dsRNA treatment. That means that RNAi technology effectively reduced gene expression. The expression level of the sdh gene after GFP dsRNA treatment was not significantly different from that of the blank control group, indicating that the effect observed in the SDH dsRNA-treated group was specific. Then, *B. xylophilus* in RNAi and the control group were exposed to cyclobutrifluram for 24 h. The mortality was evaluated, and results showed that nematodes in the *sdhb*-/*c*-/*d*-dsRNA-treatment group significantly lower mortality compared to the blank control and exogenous control groups (Figure 5b), while there was no significant difference between the *sdha*-dsRNA treatment group and the two control groups. Among them, the mortality of the *sdhc*-dsRNA treatment group was significantly lower than that of the other treatment groups.

## 3. Discussion

Cyclobutrifluram, developed by Syngenta, is a novel SDHI that has demonstrated significant activity against pathogens but has not yet been registered for controlling plant-parasitic nematodes. This study marks the first utilization of cyclobutrifluram as an alternative agent against *B. xylophilus*, providing a theoretical foundation for the sustainable and effective control of PWD.

Before implementing trunk injection as a preventive measure against PWD, it is crucial to ascertain the nematocidal activity of the substance in question. Compared with the other three agents (emamectin benzoate, abamectin, fluopyram), cyclobutrifluram had the best nematocidal activity against *B. xylophilus*, with an LC_50_ value of 0.1078 mg·L^−1^. In addition, cyclobutrifluram had a strong inhibitory effect on the fecundity. The female egg-laying rate decreased after being treated with this compound. These findings are consistent with those on *C. elegans*, in which the number of progenies reduced from 247 ± 70 to 144 ± 95 [15]. Moreover, half of the *B. xylophilus* J2 in the cyclobutrifluram-treated group failed to reach J4, which may be because the development of nematodes was either slowed or stopped in a hypometabolic condition of chemical stress [18]. It is similar to how sodium azide inhibits *C. elegans*, which inhibits cytochrome C in the electron transport chain and causes cell cycle arrest and, eventually, larval arrest [19]. Moreover, cyclobutrifluram significantly decreased the body lengths of both male and female first-generation nematodes. Several studies have shown that body lengths in *C. elegans* requires a highly conserved signal transduction pathway (cGMP-EGL-4) regulated by a member of the TGFβ family, DBL-1 [20]. We speculate that chemical signal affects the expression level of some guanylyl cyclase (GC), which, partnered with EGL-4, exerts effects on downstream functions. Moreover, cyclobutrifluram affects the reproductive system of nematodes, leading to a reduction in the number of germ cells [15], which may indirectly lead to a reduction in average body length.

Cyclobutrifluram showed excellent preventive effects on PWD in both the greenhouse and the field. Pine trees infested with drug-treated nematodes displayed initial signs of wilting on the 40th day of inoculation, with all trees exhibiting wilting symptoms by the 90th day. The disease manifested itself one month later than in the control group. And, at a higher concentration of 20 mg·cm^−1^ DBH, none of the treated trees died after a period of 24 months. Similarly, none of the EB-treated trees died after a period of 26 months, contrasting with a 33% mortality of non-treated pines [5]. EB was used to control *B. xylophilus* through GABA receptor and glutamate-gated chloride channels. Cyclobutrifluram in the present study was used to kill *B. xylophilus* by SDHI. Post-treatment, the nematodes were suppressed through the drug’s specific mechanism, leading to a reduction in the nematode population and alleviation of the disease.

In this study, we characterized *sdha* to *d* four genes expressed in *B. xylophilus*. After *sdhs*-dsRNA treatment, the egg hatching rate, locomotor capacity, and body lengths of nematodes were significantly reduced, which is similar to reported RNAi phenotypes and mutations in electron transport chain molecules of *C. elegans* [21]. These observed characteristics suggest a compromised mitochondrial function, leading to adverse effects on the nematodes’ development and behavior. This mechanism of action is perhaps what makes cyclobutrifluram so effective. RNA interference techniques are widely used to identify pesticide target genes. The sensitivity of nematodes to succinate dehydrogenase inhibitory agents has been demonstrated [22]. A resistance screen conducted on *C. elegans* revealed that specific amino acid mutations within the SDHB, SDHC, and SDHD protein subunits of complex II conferred insensitivity to the wact-11-family compound. The wact-11 family shares a core structure that is closely related to fluopyram and cyclobutrifluram [22,23]. In the current study, RNAi of *sdhb*/*c*/*d* greatly decreased cyclobutrifluram-induced mortality in *B. xylophilus*, indicating the agent’s target within the SDH complex of the mitochondrial respiratory chain. Notably, the *sdhc*-dsRNA treatment group exhibited significantly lower mortality rates compared to the other treatment groups. Molecular docking studies have further revealed that SDHC served as the primary target site for the agent on *B. xylophilus*. SDHC mutants also show resistance to cyclobutrifluram and fluopimomide in *C. elegans* [12,15]. SDHC mutants have a dysfunction of the SDH enzyme, leading to an abnormal energy metabolism with increased sensitivity to oxidative damage [24]. These SDHC mutants eliminate the competition site between the drug and lipid-soluble redox-active quinones, thus rendering the nematodes drug-insensitive. These findings suggest that the primary mode of action of cyclobutrifluram against *B. xylophilus* primarily involves its interaction with SDHC within the SDH enzyme complex.

In addition, Heydari et *al*.‘s transcriptome enrichment analysis revealed that genes encoding cytochrome P450 and UDP glucosyltransferase (UGT) were highly expressed in *C. elegans* when exposed to cyclobutrifluram [15]. Since these genes play crucial roles in metabolism and detoxification, we hypothesize that *B. xylophilus* may exhibit similar gene expression changes when exposed to cyclobutrifluram. Therefore, the next step of our study will focus specifically on the expression of these genes in *B. xylophilus*. This will help us further elucidate the mechanism of action of cyclobutrifluram and aid in the development of more effective nematocidal strategies.

## 4. Materials and Methods

### 4.1. Nematodes

The *B. xylophilus* specimens utilized in this study, labeled NXY61, were initially extracted from *Pinus massoniana* trees infected in Zhejiang province, China. They were subsequently isolated and cultured on *Botrytis cinerea* fungal mats, which were grown on 9 cm diameter potato dextrose agar (PDA) plates at 25 °C in darkness. The collection of nematodes at various synchronized stages (eggs, juveniles 2–4 (J2–J4), adults) was carried out using the methodology described by Zhou et al. [25]. Unmated J4 female and male nematodes were separately cultured on *B. cinerea* plates under the same conditions to obtain virgin adults after 24 h.

### 4.2. Chemicals

Technical grade abamectin and emamectin benzoate (95% concentration) were acquired from Zhejiang Shenghua Biok Biology Co., Ltd., Deqing, China. Fluopyram (96% concentration) was sourced from Bayer^®^ CropScience (China) Co., Ltd., Hangzhou, China. Cyclobutrifluram (95% concentration) was supplied by Syngenta Biotechnology (China) Co., Ltd., Shanghai, China. The 5% emamectin benzoate micro-emulsion (ME) formulation consisted of 50 g·L^−1^ emamectin benzoate, 300 mL·L^−1^ n-butyl alcohol, 150 mL·L^−1^ octylphenol polyoxyethylene-10, and sterile distilled water. The 1.8% abamectin ME formulation comprised 18 g·L^−1^ abamectin, 30 mL·L^−1^ methanol, 150 mL·L^−1^ toluene, 80 mL·L^−1^ emulsifier OP-10, and sterile distilled water. The 5% fluopyram emulsifiable concentrate (EC) formulation included 50 g·L^−1^ fluopyram, 10 mL·L^−1^ emulsifier OP-10, and acetone. Cyclobutrifluram was formulated as a 5% EC in DMSO.

### 4.3. Toxicity Test against B. xylophilus

The toxicities of 5% ME emamectin benzoate, 1.8% ME abamectin, 5% EC fluopyram, and 5% EC cyclobutrifluram against *B. xylophilus* were assessed using the dipping method [26]. These agents were prepared in at least six mass concentration gradients (Table S1), with one treatment for each concentration and distilled water serving as the control. Each well of a 96-well plate, pre-loaded with approximately 100 mixed-stage nematodes, received 100 μL of the pesticide solution. The plates were incubated at 25 °C, and after 24 h, the live and dead nematodes were counted. To avoid experimental errors, nematodes were washed and transferred to sterile water for 12 h. Nematodes were stimulated by shaking before counting. When nematodes were C- or L-shaped and not moving after stimulation with a fine needle, they were considered dead [27]. This procedure was repeated three times for each treatment, with three replicates each time. Mortality rates were reported as percentage corrected mortality (±standard error), and the linear regression equation for toxicity was calculated using IBM SPSS Statistics 26. The LC_20_, LC_50_, and LC_90_ values were then determined.

(1) $$\mathrm{Corrected}\;\mathrm{mortality}\;\left(\%\right)=\frac{\mathrm{Mortality}\;\mathrm{of}\;\mathrm{treatment}-\mathrm{Mortality}\;\mathrm{of}\;\mathrm{control}}{1-\mathrm{mortality}\;\mathrm{of}\;\mathrm{control}}\times 100$$

### 4.4. Effect of Four Nematicides on Reproduction

The four agents were prepared at sublethal LC_20_ concentrations to assess their effects on reproduction. Ten virgin male and female adult nematodes were randomly selected and treated with 0.5 mL of the agents for 24 h, with distilled water serving as the control. They were then cleaned and placed on *B. cinerea* plates, cultured at 25 °C in darkness. Reproduction was measured once the mycelia of *B. cinerea* in any of the Petri dishes were exhausted, approximately after 8–9 days. Nematodes were extracted using the Baermann funnel technique [28] and collected in a centrifuge tube, which was then filled to 10 mL with distilled water. The nematodes were killed with low heat before counting. A 100-μL well-mixed nematode suspension was drawn up with a pipette and placed on slides to count the numbers under a light microscope (Leica DMi1; Leica Microsystems, Wetzlar, Germany). The total volume was estimated after magnification by 100 times. This experiment was conducted three times, with three replicate plates per treatment.

### 4.5. Effect of Four Nematicides on Egg Deposition

The four agents were also prepared to assess their effects on female egg deposition. Ten pairs of virgin adult nematodes were extracted and randomly transferred to 3 cm Petri dishes with 2 mL of the agents at LC_20_ concentrations; distilled water served as the control. After mating and spawning, 36 h later, the number of eggs at the bottom of each Petri dish was counted using a light microscope. If an egg hatched into a J2 stage nematode, this count was included. The experiment was carried out three times, with three replicates per treatment. The number of eggs laid by each female in each replicate was calculated.

### 4.6. Effect of Four Nematicides on Egg Hatching Rate

To assess the effect of the four agents on egg hatching rate, eggs were collected from mixed-stage nematodes, which included a significant number of pregnant females, added to 3 cm Petri dishes. Approximately 100 eggs were laid in the dark for 1 h at 25 °C. The nematodes were then removed with a pipette, leaving only the eggs at the bottom of the dish. These eggs were treated with 2 mL of the agents at LC_20_ concentrations, while distilled water was used for the control. At 36 h post-treatment, the number of hatched eggs (J2) in each treatment group was recorded using a light microscope. The egg-hatching rate was calculated as follows:

(2) $$\mathrm{Hatching}\;\mathrm{rate}\;\left(\%\right)=\frac{J2}{\mathrm{eggs}\;+\;J2}\times 100$$

The experiment was performed three times, and each treatment was replicated three times.

### 4.7. Effect of Four Nematicides on Development Progress

The inhibitory effects of four agents at LC_20_ concentrations on the growth and development of *B. xylophilus* were analyzed, focusing on the proportion of sexually mature individuals as an indicator of nematode development. Synchronized J2 nematodes were treated with the four agents at LC_20_ concentrations for 24 h and washed and reared on *B. cinerea* in PDA culture plates, with distilled water as the control. The plates were incubated at 25 °C in darkness for 68 h, allowing the control group nematodes to reach sexual maturity. Nematodes were extracted and collected in a centrifuge tube. A 100-μL well-mixed nematode suspension containing approximately 100 nematodes was pipetted into 3 cm Petri dishes. The total number of nematodes and sexually mature individuals in each treatment group was observed and scored using a light microscope.

The sexual maturity rate was calculated using the following formula:

(3) $$\mathrm{The}\;\mathrm{sexual}\;\mathrm{maturity}\;\mathrm{rate}\;\left(\%\right)=\frac{\mathrm{The}\;\mathrm{number}\;\mathrm{of}\;\mathrm{sexually}\;\mathrm{mature}\;\mathrm{individuals}\;}{\mathrm{Total}\;\mathrm{nematodes}}\times 100$$

The experiment was performed three times, with three replicates per treatment.

### 4.8. Effect of Four Nematicides on Body Lengths of Offspring

To assess the effects of the four nematicides on offspring body lengths, assays were conducted with adult second-generation nematodes. Ten pairs of adult nematodes were treated with 0.5 mL of the agents for 24 h, then placed on *B. cinerea* mats for mating and egg laying, with distilled water added to the control. Their offspring were then extracted and collected in a centrifuge tube. Twenty sexually mature female and male adult nematodes were randomly selected, collected in a 1.5 mL centrifuge tube, and killed with low heat. A Nikon upright fluorescence microscope was used for photography, and ImageJ software (version 1.49) was used to measure body length. The experiment was repeated three times.

### 4.9. Control Efficacy of Cyclobutrifluram to B. xylophilus in the Greenhouse

The efficacy of cyclobutrifluram in inhibiting pathogenicity was tested. Three experimental groups were established. In the control group, suspensions containing 4000 nematodes were inoculated under the phloem of a 3-year-old black pine (*Pinus thunbergii* Parl) tree. In the treatment group, nematodes were treated with cyclobutrifluram at LC_20_ concentrations for 24 h before inoculation. Trees in the blank group were inoculated with distilled water. The inoculation site was the middle and upper parts of the trunk, with 10 replicates in each group. All trees were maintained in a greenhouse at 25 °C. The incidence of pine wilt was observed, and the pathogenicity of pine wood nematodes was analyzed. The infection rate of pine trees was used to assess *B. xylophilus* infections during 100 days after infection [29]. To check for the presence of *B. xylophilus* in a tree 100 days after infection, 5 cm branches from the top, middle, and bottom were randomly sampled, cut up, and placed in a funnel. The presence of *B. xylophilus* was observed with a microscope, and the number of nematodes per gram of branch was counted.

(4) $$\mathrm{Infection}\;\mathrm{rate}\;\left(\%\right)=\frac{\sum \mathrm{Number}\;\mathrm{of}\;\mathrm{infected}\;\mathrm{plants}\;\mathrm{with}\;\mathrm{symptoms}\;}{\mathrm{Total}\;\mathrm{number}\;\mathrm{of}\;\mathrm{plants}}\times 100$$

### 4.10. Preventative Effect against PWD under Field Conditions

**Figure 6:**
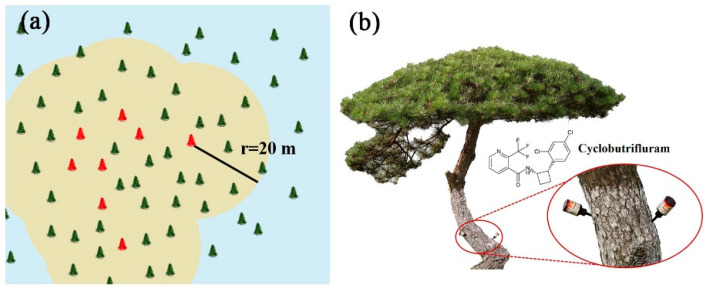
Schematic diagram of the area of trunk injection under field conditions. (**a**) The agent was injected into green trees in the infected area, defined as the field with a 20 m radius around the infected pine tree (yellow area). Trees in red are infected pine trees, while trees in green are healthy trees. (**b**) Details of the trunk injection. Holes (8 mm diameter × 7–8 cm depth) were drilled into the trunk of pine trees at a height of 1 m above ground. The enlarged part in red is the local detail map. Chemical structure of cyclobutrifluram is shown.

The “infected area” was defined as a 20 m radius around the principally PWN-infected pine trees (Figure 6a). To evaluate the preventative effect of cyclobutrifluram against PWD, healthy *P. massoniana* (18–35 cm diameter at breast height) from the infected area were used in a test in a forest at Qianlang village, Shizhu Town, Yongkang City, Zhejiang Province (28°50′ N, 120°7′ E). For trunk injections, 2% cyclobutrifluram EC and 2% emamectin benzoate ME (as a positive control) were prepared. Cyclobutrifluram was tested at 0.5 and 1.0 mL·DBH^−1^ volumes, while emamectin benzoate was tested at 1.0 mL·DBH^−1^. In the blank control groups, no agents were injected. The treatments were randomly distributed in the infected area holes (8 mm diameter × 7–8 cm depth) and were drilled into the trunks of pine trees at a height of 1 m above ground (Figure 6b). The appropriate dosages of each chemical treatment were injected into each hole using a pipette; this was performed on 10–15 June 2021, and only once. The preventative effect of each agent was determined by the mortality rate after 8, 16, and 24 months, with 41–152 trees used per treatment, and the experiment was repeated three times (Table S2). A reddish–brown coloration of all needles represents tree death. All dead trees have been felled, and parts of their trunks chopped up to analyze for the presence of the *B. xylophilus*.

(5) $$\mathrm{Mortality}\;\mathrm{rate}\left(\%\right)=\frac{\mathrm{The}\;\mathrm{number}\;\mathrm{of}\;\mathrm{dead}\;\mathrm{trees}}{\mathrm{The}\;\mathrm{number}\;\mathrm{of}\;\mathrm{treated}\;\mathrm{trees}}\times 100$$

### 4.11. Phylogenetic Analysis, Model Building, and Molecular Docking of SDHs

The amino acid sequence of the *B. xylophilus* SDH subunits (SDHA: CAD5235524.1, SDHB: CAD5215503.1, SDHC: CAD5222053.1, SDHD: CAD5221188.1) were retrieved from NCBI. Phylogenetic analysis of the SDHs in *B. xylophilus* with the amino acid sequences of this protein from other species was performed using MAGA software (version 11.0.10). A 3D structural model of *B. xylophilus* SDHs was developed by homology modeling performed by the AlphaFold2 [30]. The protein is a transmembrane complex comprising four protein subunits (SDHA to D). The experiment of SDH-autodocking with cyclobutrifluram was performed on http://hdock.phys.hust.edu.cn/ (accessed on 3 February 2024) [31].

### 4.12. Gene Clone of Four sdhs

*B. xylophilus* of developmental stages were collected, and their total RNAs were extracted. They were converted to cDNA for gene cloning and qPCR. Four *sdh* genes (*sdha*, *sdhb*, *sdhc*, and *sdhd*) encode four nuclear-encoded subunits form the SDH complex in *B. xylophilus*. Their cloning primers are designed (Table S3) and cloned into a pGEM-T Easy vector (Promega, Madison, WI, USA). Four genes were cloned to generate dsRNA for RNAi.

### 4.13. The Relative Expression Levels of sdhs Analysed by RT-qPCR

Primers for the reference gene *β-actin* (EU100952.1) and target genes of *B. xylophilus* were used in qPCR to examine the expression levels (Table S3). RT-qPCR was carried out using the qTOWER 2.2 qPCR System (Analytik JenaAG, Thuringia, Germany) with TB Green^®^ Premix Ex Taq II™ (TaKaRa, TliRNaseH Plus, Kusatsu, Japan). The relative *sdhs* gene expression data were analyzed using the 2^−ΔΔCT^.

### 4.14. dsRNA Synthesis and RNAi

T7 promoter sequences were added to the end of target genes’ cloning primers for the synthesis of dsRNA (Table S3). The green fluorescent protein-encoding gene (*gfp*, M62653.1) was used as the nonendogenous control. Four *sdh* genes and GFP dsRNA fragments were synthesized using the MEGAscript^®^ T7 High Yield Transcription Kit (Thermo Fisher Scientific Inc., Waltham, MA, USA). Nematodes were soaked in RNAi solution for 24 h, which contained 4 μL of M9 buffer, 10 μg *sdha/b/c/d*-dsRNA, and ddH_2_O. Nematodes were soaked in M9 buffer without dsRNA as blank control. Nematodes were soaked with *gfp*-dsRNA as exogenous control. All treatment solutions were up to 20 μL; each treatment had three replicates.

Virgin male and female nematodes were treated with different RNAi solutions for 24 h. For each group, 10 pairs of nematodes were selected for mixed culture, and the number of eggs laid by each female was counted after 36 h. After 24 h, ten hatched J2s were randomly selected from each group, and their motility was evaluated in terms of head swing frequency per minute. J2 was transferred to a *B. cinerea* mat and cultured into an adult. Ten male nematodes were selected, and their body lengths were measured. Each treatment had three replicates.

### 4.15. The Sensitivity of B. xylophilus to SDHs

The mixed-age *B. xylophilus* were cultured and collected, treated by *sdh*-dsRNA for 24 h in the treatment group, treated with *gfp*-dsRNA in the exogenous control group, and treated with ddH_2_O in the blank control group. The total RNA of mixed-age nematodes in each treatment group was extracted and reverse-transcribed into cDNA. The RNA interference efficiency of *Bxy-sdh*-dsRNA was detected by RT-qPCR. The other mixed-age nematodes in each treatment group were exposed to LC_20_, LC_50_, LC_90_ (0.031, 0.1078, 0.7211 mg/L) of cyclobutrifluram using the toxicity test method, as described above. Mortality was evaluated after treatment with cyclobutrifluram for 24 h. This experiment was conducted three times, with approximately 100 nematodes per treatment.

### 4.16. Data Analysis

All statistical analyses were conducted using Microsoft Excel 2019, and the data were expressed as mean ± standard error (SE). GraphPad Prism 8 was used for the homogeneity test of variance and one-way analysis of variance (ANOVA), and multiple comparisons were performed using Tukey’s (HSD) test (*p* = 0.05) to analyze the significant difference.

## 5. Conclusions

*B. xylophilus* is a dangerous quarantine pest that damages pine ecosystems worldwide. Cyclobutrifluram, introduced by Syngenta in 2013, shows high efficacy against *B. xylophilus*, significantly reducing its population and preventing PWD. This study confirms that SDHC is the target, highlighting cyclobutrifluram’s potential for trunk injection to control and prevent pine wilt disease in forests.

## Figures and Tables

**Table 1:** Toxicity results of the four agents against *B. xylophilus*.

Treatments	LC_20_ ^a^ (95% CI) ^b^	LC_50_ (95% CI)	LC_90_ (95% CI)	Slope ± SE ^c^	χ^2^
Cyclobutrifluram 5% EC	0.0310 (0.0229–0.0394)	0.1078 (0.0883–0.1333)	0.7211 (0.5065–1.1573)	1.5530 ± 0.0388	69.2864
Emamectin benzoate 5% ME	0.0420 (0.0170–0.1287)	0.2783ta (0.1721–0.4156)	4.9635 (2.8710–11.0051)	1.0243 ± 0.0269	81.1801
Abamectin 1.8% ME	0.0890 (0.0311–0.1876)	1.3874 (0.8023–2.2188)	90.9167 (45.7726–236.2216)	0.7055 ± 0.0195	54.4470
Fluopyram 5% EC	0.4263 (0.2892–0.5737)	1.4867 (1.1950–1.8011)	9.9628 (7.7086–13.8221)	1.5512 ± 0.0391	51.1241

^a^ LC_20_, LC_50_, and LC_90_ (mg/L) data were calculated after 24 h of treatment with Cyclobutrifluram 5%. ^b^ 95% confidence interval. ^c^ The equation of linear regression for toxicity was estimated using IBM SPSS Statistics 26; then, the slope of equation was obtained.

**Table 2:** Preventative effect of emamectin benzoate and cyclobutrifluram against PWD under field conditions ^a^.

Chemical	Injection Volume (mL DBH^−1^) ^b^	Mortality Rate (Mean ± SE, %)		
		8 Months ^c^	16 Months	24 Months
Blank control	-	3.38 ± 0.46 ^a,d^	10.74 ± 3.28 ^a^	26.52 ± 2.74 ^a^
2% Emamectin benzoate ME	1	0.44 ± 0.36 ^b^	1.34 ± 0.17 ^b^	2.14 ± 0.58 ^b^
2% Cyclobutrifluram EC	0.5	0.26 ± 0.21 ^b^	0.52 ± 0.43 ^b^	1.27 ± 0.77 ^b^
	1.0	0 ± 0.00 ^b^	0 ± 0.00 ^b^	0 ± 0.00 ^b^

^a^ The preventative effect of each agent was determined by using mortality rate of *P. massoniana*. ^b^ Injection volume (mL DBH^−1^): the volume of substance (in milliliters) injected per unit of Diameter at Breast Height (DBH) of a tree. ^c^ The mortality rate of pine trees after 8, 16, and 24 months of drug treatment was calculated, with 41–152 trees used per treatment, and the experiment repeated three times. ^d^ Results in the same column with the same letter did not show significant differences (*p* < 0.05).

## Data Availability

The datasets are available from the corresponding author upon reasonable request.

## Supplementary Materials

The following supporting information can be downloaded at https://www.mdpi.com/article/10.3390/ijms25136914/s1.

## Abbreviations

Succinate dehydrogenase inhibitor (SDHI); pine wood nematode (PWN); pine wilt disease (PWD); potato dextrose agar (PDA); standard error (SE); analysis of variance (ANOVA); guanylyl cyclase (GC); micro-emulsion (ME).
