# Supplementary material for: The New Nematicide Cyclobutrifluram Targets the Mitochondrial Succinate Dehydrogenase Complex in Bursaphelenchus xylophilus
Source: Int J Mol Sci. 2024 Jun 24;25(13):6914. doi: 10.3390/ijms25136914 (PMC11241274; doi:10.3390/ijms25136914)
Supplement: Supplementary file 1 [file ijms-25-06914-s001.zip › ijms-3047699-supplementary.pdf]

Supporting Information

The New Nematicide Cyclobutrifluram Targets the Mitochondrial Succinate Dehydrogenase Complex in *Bursaphelenchus xylophilus*

Wenyi Liu [1†], Hudie Shao [1†], Danni Qi [1], Xiaofang Huang [1], Jing Chen [1], Lifeng Zhou [1], and Kai Guo [1*]

[1] College of Forestry and Biotechnology, Zhejiang A & F University, Hangzhou 311300, China.

[†] Wenyi Liu and Hudie Shao contributed equally to this study.

[*] Correspondence to: Kai Guo
Email address: kaiguo@zafu.edu.cn (Guo)

Table S1. Corrected mortality rates of the four agents: emamectin benzoate 5% ME, abamectin 1.8% ME, fluopyram 5% EC, and cyclobutrifluram 5% EC, against *B. xylophilus*

Chemical	Concentration ($mg \cdot L^{-1}$) [a]	Corrected mortality±SE (%) [b]
Cyclobutrifluram 5% EC	0.0078125	9.17±1.11 g [c]
	0.015625	12.29±1.01 g
	0.03125	19.45±0.29 f
	0.0625	28.24±1.23 e
	0.125	53.67±1.55 d
	0.25	73.16±1.09 c
	0.5	81.75±0.67 b
	1	97.33±0.99 a
Emamectin benzoate 5% ME	0.03125	10.54±0.98 f
	0.125	38.79±0.80 e
	0.25	57.29±0.63 d
	1	68.42±1.68 c
	4	84.24±0.81 b
	8	95.83±1.05 a
Abamectin 1.8% ME	0.125	19.47±0.66 f
	0.25	35.60±0.33 e
	2	52.68±0.97 d
	16	71.60±0.86 c
	32	82.92±1.04 b
	64	92.51±0.32 a
Fluopyram 5% EC	0.3125	18.83±0.83 g
	0.625	26.22±1.30 f
	1.25	36.21±0.81 e
	2.5	69.89±0.70 d
	5	78.63±1.74 c
	10	89.76±0.81 b
	20	96.23±0.75 a

[a] The agents were prepared into at least six mass concentration gradients against mixed-stage *B. xylophilus* for 24 h using the dipping method, with one treatment for each concentration, and distilled water as the control.

[b] The toxicity of each of the four agents was measured and recorded with the corresponding death rates, based on the different concentrations of nematicide.

[c] Different letters indicate that the difference has reached a significant level between two groups ($P<0.05$).

Table S2. Preventative effect of EB and cyclobutrifluram against PWD under field conditions [a]

Chemical	Injection volume (mL DBH^{-1}) [b]	Number of deaths/total trees [c]								
		8 months			16 months			24 months		
		1 [d]	2	3	1	2	3	1	2	3
Blank control	-	2/48	3/131	5/136	9/48	8/131	10/136	14/48	40/131	27/136
2% Emamectin benzoate ME	1	0/58	0/100	2/152	1/58	1/100	2/152	2/58	1/100	3/152
2% Cyclobutrifluram EC	0.5	0/41	0/147	1/128	0/41	0/147	2/128	0/41	1/147	4/128
	1.0	0/45	0/147	0/129	0/45	0/147	0/129	0/45	0/147	0/129

[a] The preventative effect of each agent was determined by using mortality rate of *P. massoniana* after treatment.

[b] DBH: diameter at breast height (cm).

[c] The preventative effect of each agent was determined by the mortality rate after 8, 16 and 24 months.

[d] 41–152 trees were used per treatment and the experiment repeated three times.

Table S3. PCR primers used in the current study.

Primer	Gene symbol	Sequence (5′–3′)
Cloning	*Bxy-sdha*	F: TTCATGGAGAGATACGCGCC<br>R: CGTCGATGACGGGTCTGTAG
	Bxy-sdhb	F: CGGCGGCAGTTTTATTCGTT<br>R: TCGAGGCCAGTCAGCAATTT
	Bxy-sdhc	F: TCGCCAATTCAAAACCAGCG<br>R: GCCAAGTCAAAGCCAACGAG
	Bxy-sdhd	F: CTTCCGTACTTTCGCCCCTT<br>R: AAAGGGCCGTTCCAACGTAT
RT-qPCR	*Bxy-sdha*	F: CTGTTTGATGACCGAGGGCT<br>R: TGACGGGGATCGGTTCTTTG
	Bxy-sdhb	F: GTGGCAATGTCCGGATGAGA<br>R: ACGAAAGGCTTGAACCGAGT
	Bxy-sdhc	F: TGAAACGGCCTATTGCACCT<br>R: CCAACGAGTCTCAGGCCATT
	Bxy-sdhd	F: AGAGCCTTCCGTACTTTCGC<br>R: CGGACGGGCATAATCTGTGA
	Bxy-β-actin	F: CGCAAATACTCCGTCTGGATTGG<br>R: TTCGTCGTACTCTTGCTTGGAGA
Synthesis of dsRNA	T7 promoter	TAATACGACTCACTATAGGG
	gfp	F: TAATACGACTCACTATAGGGAAAGGAGAAGAACTTTTCAC<br>R: TAATACGACTCACTATAGGGCTGTTACAAACTCAAGAAGG
